# Positive Association between Tinnitus and Arterial Hypertension

**DOI:** 10.3389/fneur.2016.00171

**Published:** 2016-10-05

**Authors:** Ricardo Rodrigues Figueiredo, Andréia Aparecida Azevedo, Norma De Oliveira Penido

**Affiliations:** ^1^Otolaryngology, Universidade Federal de São Paulo, São Paulo, Brazil; ^2^Otolaryngology, Faculdade de Medicina de Valença, Valença, Brazil

**Keywords:** tinnitus, arterial hypertension, hearing loss, cardiovascular diseases, hearing disorders

## Abstract

**Introduction:**

Tinnitus is the perception of noise in the absence of an external source and is considered by most authors as a multifactorial symptom. A systematic review concerning the association of tinnitus and systemic arterial hypertension retrieved suggestions of a positive association, but the articles included failed to perform a detailed analysis on the theme.

**Purpose:**

To analyze the presence of arterial hypertension in tinnitus and non-tinnitus patients, to analyze differences between tinnitus impact and psychoacoustic measurements in hypertensive and normotensive patients, and to evaluate the association between the presence of tinnitus and the diverse antihypertensive drugs employed.

**Materials and methods:**

This includes cross-sectional transversal study, comparing two groups of subjects (144 in the study group with tinnitus and 140 in the control group without tinnitus). Clinical, demographical, audiometrical, and psychoacoustics characteristics of the subjects were compared.

**Results:**

Hypertension prevalence in tinnitus subjects was 44.4% against 31.4% in subjects without tinnitus (*p* = 0.024). Positive associations with tinnitus were found with hypertension treatment with angiotensin-converting enzyme (ACE) inhibitors (*p* = 0.006), tiazidic diuretics (*p* < 0.0001), potassium-sparing diuretics (*p* = 0.016), and calcium channels blockers (*p* = 0.004).

**Conclusion:**

There is an association between tinnitus and arterial hypertension. This association is particularly strong in older patients. Hypertension treatment with diuretics, ACE inhibitors, and calcium channels blockers were more prevalent in tinnitus patients, suggesting that an eventual ototoxicity of these drugs may be involved in tinnitus pathophysiology, a hypothesis that should be evaluated in further studies.

## Introduction

Tinnitus is the perception of noise, which is not generated by external stimulus (1). It affects approximately 25% of the general population: one third on a frequent basis (2). Tinnitus may be classified as auditory and para-auditory tinnitus with the former representing the majority of cases, and the latter being subdivided into muscular and vascular tinnitus, sometimes referred as somatosounds (3).

According to the most recent trends of thought, tinnitus is considered a symptom which may have multiple causes, sometimes even in a single patient (4, 5). Noise exposure, metabolic and cardiovascular disease, presbycusis, ototoxicity, and cranial and cervical trauma are the most frequently considered causes of tinnitus (5, 6). Caffeine abuse, dietary factors, temporomandibular joint, and cervical disease have also been described as contributing factors (7–9).

Systemic arterial hypertension is a multifactorial clinical condition characterized by raised and sustained arterial pressure levels (10). It is defined as systolic levels equal or greater than 140 mmHg and diastolic levels equal or greater than 90 mmHg (10). The prevalence in Brazil, similar to that in other countries, is estimated to be between 22.3 and 43.9% (32.5% average), raising to 50% between 60 and 69 years old and 75% for 70 years and older (10–12). The presence of comorbidities, such as diabetes mellitus and dyslipidemias, and habits, such as smoking, was demonstrated to increase complications risks (13). Arterial hypertension has been described as a possible cause of tinnitus since 1940s (14). Three principle mechanisms suspected of being involved are: damage to inner ear microcirculation (15), ototoxicity by antihypertension drugs (16), and perception of noise generated by blood vessels (3).

As related to inner ear microcirculation, the *stria vascularis* was demonstrated to be the main cochlear site damaged by arterial hypertension (17). Sodium retention could also lead to an increase of extracellular fluid volume, including the perilymph (18), and the endocochlear potential being reduced in hypertensive rats (19). Moreover, hypertension has been associated with a higher risk of hearing loss in brain ischemia (15) and also with a slower recovery in sudden hearing loss (20).

In considering ototoxicity, an extensive review cited diuretics, beta-blockers, angiotensin-conversing enzyme (ACE) inhibitors, angiotensin II receptors blockers, and calcium channels blockers as possible ototoxic medications (21). Furosemide’s ototoxicity is the most studied form, producing a quick and reversible decrease of the endocochlear potential (22).

As for vascular tinnitus, some studies cite hypertension as a causal factor, mainly when vascular abnormalities have been ruled out (3). An anatomopathological study demonstrated a high incidence of bony dehiscence of the carotid canal in the middle ear, which may affect the inner ear microcirculation and also generate vascular noises (23).

According to a systematic review, there is evidence of an association between tinnitus and arterial hypertension, but there is a lack of more comprehensive studies (24). The association is stronger in studies that analyzed the presence of arterial hypertension in patients with tinnitus than in those which analyzed the presence of tinnitus in patients with arterial hypertension.

The main purpose of this study is to analyze the presence of arterial hypertension in tinnitus and non-tinnitus patients. Secondary purposes are to analyze differences between tinnitus impact and psychoacoustic measurements in hypertensive and normotensive patients and to evaluate the association between the presence of tinnitus and the diverse antihypertensive drugs employed.

## Materials and Methods

This is a transversal case–control study in which individuals of 18 years of age or older with and without tinnitus were selected at the author’s ENT clinic from 2011 to 2014. The trial was approved by the Institutional Review Board (number 010/CEP-FMV/2011). This study was carried out in accordance with the recommendations of the aforementioned Institutional Review Board with written informed consent from all subjects. All subjects gave written informed consent in accordance with the Declaration of Helsinki.

Two groups were created: the first included patients with tinnitus of at least 3 months duration and the second included patients without tinnitus (control). The control group was paired with the tinnitus group for gender, age, and race. The time of tinnitus onset as related to arterial hypertension onset was not an exclusion criteria. Patients from both groups were submitted to anamnesis (including demographics, comorbidities, and habits), otorhinolaryngological physical examination, and arterial pressure measurements with a calibrated sphygmomanometer (Erka Perfekt Aneroid, Germany), in order to exclude possible undiagnosed arterial hypertension. The criteria for blood pressure evaluation were those from the VII Joint National Committee on Prevention, Detection, Evaluation, and Treatment of High Blood Pressure, U. S. Department of Health and Human Services, as previously described.

Patients allegedly normotensive with high blood pressure detected at the physical examination were excluded. Patients from both groups also underwent conventional pure tone and speech audiometry.

Tinnitus patients were questioned regarding their tinnitus characteristics (duration, type of sound, laterality, and periodicity) and also classified their tinnitus according to a Visual Analog Scale (VAS), from 1 to 10 (for intensity and distress) and to the Brazilian Portuguese validated version of the Tinnitus Handicap Inventory (THI) (25). They also underwent psychoacoustic measurements of their tinnitus – Pitch Matching (PM) and Minimum Masking Level (MML).

The sample size was determined after the analysis of the arterial hypertension prevalence in a preliminary sample of tinnitus patients (*n* = 46) that was compared to the prevalence of arterial hypertension in the Brazilian general population, obtained from previous studies (10). The minimum number of individuals for each group was determined to be 140.

For whole sample analysis, the comparison of the variables between the tinnitus group and the control group was performed using the Mann–Whitney test for the numerical data and the chi-square or the Fisher test for the categorical data. The Spearman coefficient was performed to analyze the association between the duration of arterial hypertension and the numerical audiometry measurements. The Cochran–Mantel–Haenszel was used to analyze the association between tinnitus and arterial hypertension adjusting for the presence of hearing loss. The significance level was established at 5%, and the statistical analysis was performed with the SAS statistical software, 6.11 version (SAS Institute, Inc., Cary, NC, USA).

## Results

The final sample was composed of 144 patients in the tinnitus group and 140 in the control group. The tinnitus group was then divided into two subgroups: one for patients with arterial hypertension and the other without arterial hypertension.

The average age was 57.8 years of age for the tinnitus group and 58.6 for the control group. The averages of tinnitus duration, VAS for intensity, VAS for distress, and THI scores for the tinnitus group were, respectively, 54.5 months–4.5 years, 5.45, 5.83, and 41.7 points.

Although the duration of arterial hypertension was longer (average of 128.7 months–10.7 years) than the duration of tinnitus (average of 54.5 months), no statistically significant correlation was found, according to the Spearman coefficient. Considering the patients that could ascertain the duration of both arterial hypertension and tinnitus (*n* = 56), for 42 of them (75%) arterial hypertension preceded tinnitus.

Table 1 shows the comparison of numerical variables and Table 2 shows the categorical variables, the latter confirming the pairing by gender, age, and race. As shown in Table 2, arterial hypertension was more prevalent in tinnitus patients (*p* = 0.024), demonstrating an association of tinnitus and arterial hypertension. This association was not found when data were adjusted for the presence of hearing loss (*p* = 0.27, according to the Cochran–Mantel–Haenszel test). Hearing loss was more frequent in the tinnitus group, as shown in Table 3, but there was no difference between the two groups as to the type of hearing loss or the shape of the audiogram curve. Sensorineural descendant was the most frequent curve in both groups (75% in the tinnitus patients group and 58% in the control group). Also, there was no difference in the speech recognition index of both groups (100% median in both).

**Table 1:** **Analysis of the numerical variables (age, duration of arterial hypertension, and daily consumption of caffeine) according to the group**.

Variable	Tinnitus			No tinnitus			*p*-Value
	*n*	Median	IQA	*n*	Median	IQA	
Age (years)	144	59	49–69	140	58	50–67	0.97
Arterial hypertension duration (months)	60	**120**	39–216	43	**180**	84–240	**0.019**
Caffeine (ml/day)	144	**100**	50–200	140	**300**	200–400	**0.0001**

*Significant values in bold*.

*IQA, Interquartilic Amplitude: Q1–Q3*.

**Table 2:** **Analysis of categorical variables (gender, age, race, and presence of arterial hypertension) according to the groups**.

Variable	Category	Tinnitus		No Tinnitus		*p*-Value^a^
		*n*	%	*n*	%	
Gender	Male	62	43.1	65	46.4	0.57
	Female	82	56.9	75	53.6	
Age (years)	≤40	18	12.5	14	10.0	0.82
	41–59	55	38.2	60	42.9	
	60–69	40	27.8	36	25.7	
	≥70	31	21.5	30	21.4	
Race	White	93	71.5	98	70.0	0.94
	Brown	22	16.9	24	17.1	
	Black	15	11.5	18	12.9	
Arterial Hypertension	Yes	64	**44.4**	44	**31.4**	**0**.**024**
	No	80	55.6	96	68.6	

*Significant values in bold*.

*^a^χ^2^ or Fisher test*.

**Table 3:** **Prevalence of hearing loss among tinnitus and non-tinnitus patients (χ^2^ test)**.

Variable	Category	Tinnitus		No Tinnitus		*p*-Value
		*N* (ears)	%	*N* (ears)	%	
Hearing loss	Yes	111	81.3	75	53.6	<0.0001
	No	32	18.7	65	46.4	

The analysis of the antihypertensive drugs used in both groups is shown in Table 4.

**Table 4:** **Analysis of the categorical variable – antihypertensive drugs used according to the groups**.

Variable	Category	Tinnitus		No tinnitus		*p*-Value
		*n*	%	*n*	%	
B-blocker	Yes	19	13.2	21	15.0	0.66
	No	125	86.8	119	85.0	
ACEI	Yes	23	16.0	8	5.7	0.006
	No	121	84.0	132	94.3	
ARB	Yes	34	23.6	24	17.1	0.18
	No	110	76.4	116	82.9	
Loop diuretic	Yes	0	0.0	4	2.9	0.057
	No	144	100.0	136	97.1	
Thiazidic diuretic	Yes	29	20.1	8	5.7	<0.0001
	No	115	79.9	132	94.3	
K sparing diuretic	Yes	6	4.2	0	0.0	0.016
	No	138	95.8	140	100.0	
CCA	Yes	13	9.0	2	1.4	0.004
	No	131	91.0	138	98.6	

*χ-squared or Fisher tests*.

*ACEI, angiotensin-converting enzyme inhibitor; ARB, angiotensin II receptor blocker; CCA, calcium channel antagonist*.

The analysis of comorbidities and habits demonstrated that dyslipidemia was more frequent in the tinnitus group (*p*-value of 0.003), while the presence of diabetes mellitus, hypothyroidism, noise exposure, caffeine consumption, and smoking was similar in both groups. When considering the coexistence of arterial hypertension and comorbidities and habits, the association of arterial hypertension and caffeine consumption greater than 150 milliliters per day was more frequent in the tinnitus group (*p* < 0.0001).

Table 4 shows data about the antihypertensive drugs used in both groups.

Table 5 shows data from the numerical variables for the tinnitus’ patients subgroups, those with and without arterial hypertension.

**Table 5:** **Analysis of the numerical variables (age, caffeine consumption, tinnitus duration, Visual Analog Scale, Tinnitus Handicap Inventory, Pitch Masking, and Minimum Masking Level) according to the subgroups of tinnitus patients (with and without arterial hypertension)**.

Variable	Arterial hypertension subgroup			No arterial hypertension subgroup			*p*-Value^a^
	*n*	Median	IQA	*n*	Median	IQA	
Age (years)	64	**66**	57–72	80	**52.5**	43–65	**0.0001**
Caffeine (ml/day)	58	100	50–200	67	100	100–200	0.78
Tinnitus duration (months)	59	24	9–60	76	18	5.3–60	0.22
VAS volume (points)	63	5	3–7	80	6	4–8	0.37
VAS distress (points)	63	5	3–8	80	6	4–9	0.37
THI score (points)	63	40	18–70	80	38	16.5–61.5	0.75
PM RE (Hz)	40	4000	1000–8000	64	6000	2000–8000	0.58
PM LE (Hz)	45	4000	1500–8000	56	6000	3250–8000	0.27
MML RE (dB SL)	40	15	5–25	63	15	10–30	0.36
MML LE (dB SL)	44	15	5–29	55	15	5–25	0.48

*Nineteen patients reported no caffeine consumption, nine patients could not estimate the duration of their tinnitus, and one patient could not report VAS and THI scores*.

*IQA, interquartilic amplitude: Q1–Q3*.

*Bold is to highlight the significant data*.

*^a^Mann–Whitney test*.

*VAS, Visual Analog Scale; THI, Tinnitus Handicap Inventory; PM, Pitch Masking; MML, Minimum Masking Level; RE, right ear; LE, left ear; dB, decibel; dB SL, decibel Sensation Level*.

There was no statistical difference between the two subgroups concerning gender and race. Diabetes mellitus and dyslipidemia were more frequent in the subgroup with tinnitus and arterial hypertension (*p* = 0.017 and 0.02, respectively), while there were no differences concerning hypothyroidism, noise exposure, caffeine consumption, or smoking. Hearing loss was considered to be more prevalent in the subgroup with arterial hypertension (89.1%), although it was also frequent in the normotensive subgroup (75%, *p* = 0.032). No differences concerning the type or curve were found.

Table 6 shows the tinnitus characteristics in both subgroups.

**Table 6:** **Analysis of the categorical variables (tinnitus clinical characteristics) according to the tinnitus’ patients’ subgroups (with and without arterial hypertension)**.

Variable	Category	Arterial hypertension subgroup		No arterial hypertension subgroup		*p*-Value^a^
		*n*	%	*n*	%	
Laterality	RE	13	20.3	22	27.5	0.60
	LE	15	23.4	14	17.5	
	Bilateral	32	50.0	41	51.3	
	Head	4	6.3	3	3.8	
Instalation	Sudden	33	55.0	33	42.9	0.0.16
	Gradual	27	45.0	44	57.1	
Periodicity	Constant	45	70.3	55	69.6	0.93
	Intermittent	19	29.7	24	30.4	

*^a^χ^2^ or Fisher test*.

*RE, right ear; LE, left ear*.

The most frequently described types of tinnitus were wheezing, whistle, and insect with no differences between the subgroups. The presence of multiple types of sound in the same patient was more frequently found in the hypertensive subgroup (*p* = 0.014). The prevalence of vascular tinnitus was 6.3% in the hypertensive subgroup and 1.3% in the normotensive one (*p* = 0.12) with muscular tinnitus being found in only two cases, both being in normotensive patients.

No significant differences were found between the subgroups concerning the otolaryngologic exam, including evaluation of the temporomandibular joint. Finally, there was no correlation discovered between the duration of arterial hypertension and tinnitus according to the Spearman coefficient (*p* = 0.77).

## Discussion

Association studies are key sources of information for the comprehension of how one disorder may affect another. A study on attempt to establish a correlation between arterial hypertension and tinnitus studies will help to elucidate characteristic in common that could be addressed in the diagnosis and therapeutic interventions. However, causal correlate studies would demand a plethora of different cases, including patients without tinnitus or arterial hypertension who were also free from other infirmities or habits which could be linked to tinnitus generation. Oftentimes, this elaborate type of study requires an extensive time period and patients with incredibly varied conditions.

A systematic review on this subject (24) argued that studies which have evaluated the prevalence of tinnitus among hypertensive patients failed to demonstrate an association (26–29). However, data from this study, along with other studies (2, 30, 31), demonstrated an association between tinnitus and arterial hypertension (hypertension prevalence in tinnitus patients = 44.4% against 31.4% in patients without tinnitus, *p* = 0.024).

The information above may lead one to infer that tinnitus could be a causal factor for hypertension, albeit it seems more reasonable to believe arterial hypertension is more a cofactor than a main cause of tinnitus.

Regarding hearing loss, the results are in conformity with prior studies, which found a high prevalence among tinnitus patients, as well as the higher prevalence of sensorineural pattern with descending configuration curves (2, 5).

Once hearing loss was added to (adjusted for) the statistical analysis, the association between tinnitus and arterial hypertension was no longer positive, suggesting that arterial hypertension may be a cause of hearing loss, which is related to most cases of tinnitus, as previously reported (1, 6). Having said so, if we think about tinnitus prevention, arterial hypertension may still be regarded as a possible important causal factor.

Arterial hypertension may affect the inner ear microcirculation, and it is known that comorbidities, such as diabetes mellitus and dyslipidemia, may enhance vascular impairment due to hypertension (10). Although dyslipidemia was more prevalent in the tinnitus group, there was no difference concerning the concomitancy of arterial hypertension and dyslipidemia. Diabetes mellitus also affects the inner ear microcirculation, but it may also have direct metabolic effects on cochlea (4, 5, 20). There was a statistical tendency in favor of a higher prevalence of the concomitancy of diabetes and hypertension in the tinnitus group. As for hypothyroidism, another metabolic disease frequently cited as related to tinnitus (6), no differences between the two groups were found, either as an isolated factor or in association with arterial hypertension. The comparison between the tinnitus subgroups (with and without hypertension) demonstrated that diabetes mellitus and dyslipidemia were more prevalent in hypertensive tinnitus patients, suggesting that these diseases may act synergically upon the generation, maintenance and/or aggravation of tinnitus.

The excessive consumption of caffeine is believed to have a negative impact on hypertension control (32), but its association with tinnitus has been doubted by recent studies (9, 33, 34). Data from this study failed to demonstrate any association between tinnitus and caffeine consumption. In fact, the concomitancy of hypertension and caffeine intake greater than 150 ml per day was more prevalent in the control group, which may reflect the widespread patients’ concept that caffeine worsens tinnitus. Smoking, which is believed to worsen both tinnitus and hypertension, probably by impairing macro and microcirculation (10, 35), was more prevalent in the tinnitus group, either as an isolated factor or concomitant to hypertension.

Considering these findings, one might speculate that vascular changes, which may affect cochlear microcirculation, leading to hair cells damage and, consequently, tinnitus is probably a common pathophysiological scenario for many conditions, such as arterial hypertension, dyslipidemia, diabetes mellitus, smoking, and caffeine abuse. These conditions are not infrequently found in a single patient and may act sinergically, multiplying the damage to the auditory system.

Although noise exposure is considered as one of the main causes of tinnitus (4, 6), data from this study failed to establish an association between noise exposure and tinnitus, either isolated or concomitant to hypertension. This finding should be considered with caution, being that the study was performed in an industrial city where many workers are exposed to industrial or recreational noise.

The average age of patients with tinnitus and arterial hypertension was significantly higher than the age of those in the group with tinnitus and no arterial hypertension. In the hypertension tinnitus subgroup most of the patients were 60 years or older, while the opposite was verified in the subgroup without hypertension. Both tinnitus and arterial hypertension are more prevalent in the elderly (2, 10), but these findings may also be due to some synergistic action of presbycusis and arterial hypertension contributing to tinnitus generation and, eventually, aggravation.

The difference concerning arterial hypertension duration, which was significantly lower in the tinnitus group, may be due to a lack of proper control of blood pressure in the first years, which may lead to perfusion and reperfusion vascular events in the cochlea. More studies are needed to clarify these findings.

The median of the pitch masking was higher (6 kHz) in the subgroup without hypertension than when compared to the hypertension subgroup (4 kHz), although this difference was not statistically significant. Both measures are in agreement with most of the references, tinnitus ranging from 3 to 8 kHz, which corresponds to the usually most affected frequencies in hearing loss (36, 37). As for the MML, the averages of both subgroups were the same (15 dB SL), which is somewhat higher than the usually reported sensation level of 5–10 dB SL (37). No differences were demonstrated concerning the degree of intensity and distress due to tinnitus in both subgroups, considering the VAS and the THI.

The tinnitus’ characteristics were similar in both groups, including the type of sound perceived by the patients. The fact that multiple sounds’ tinnitus was more prevalent in the hypertensive patients may reflect the multicausality of tinnitus, hypertension being one of the possible factors involved.

Although the average duration of arterial hypertension was longer than the duration of tinnitus and for most of the patients the onset of hypertension preceded the onset of tinnitus, the methodology of this case–control does not allow the conclusion that hypertension is a causal factor for tinnitus.

The ototoxicity of many antihypertension medications has been well established, especially with diuretics (21, 22). Data from this study demonstrated that the use of ACE inhibitors, thiazidic diuretics, potassium-sparing diuretics, and calcium channels blockers was more prevalent in the tinnitus hypertensive patients than in the control group. These findings have a partial correspondence with prior studies (16) and, although they are not strong enough to justify a correlation between an eventual ototoxicity of these drugs and the presence of tinnitus (for example, multidrug therapy for hypertension is very frequent), it appears that further, more detailed studies on this subject should be performed.

## Conclusion

There is an association between tinnitus and arterial hypertension. This association is particularly strong in older patients and cannot be dissociated from the hearing loss, which was also more prevalent among tinnitus patients. The use of thiazidic and potassium-sparing diuretics, ACE inhibitors, and calcium channels blockers was more prevalent in tinnitus patients.

The clinical and psychoacoustic characteristics of tinnitus in hypertensive and normotensive patients were similar, as well as tinnitus-related distress.

## Author Contributions

RF – study design, data collection, and writing. AA – data collection. NP – study design and writing.

## Conflict of Interest Statement

The authors declare that the research was conducted in the absence of any commercial or financial relationships that could be construed as a potential conflict of interest.
